# Synergistic effects of cold atmospheric plasma and doxorubicin on melanoma: A systematic review and meta-analysis

**DOI:** 10.1038/s41598-025-90508-z

**Published:** 2025-03-06

**Authors:** Zeinab Rostami, Reza Alizadeh-Navaei, Monireh Golpoor, Zahra Yazdani, Alireza Rafiei

**Affiliations:** 1 Department of Immunology, School of Medicine, Mazandaran University of Medical Sciences, KM 18 Khazarabad Road, KhazarSq, Sari, Iran; 2 Student Research Committee, School of Medicine, Mazandaran University of Medical Sciences, Sari, Iran; 3 Gastrointestinal Cancer Research Center, Mazandaran University of Medical Sciences, Sari, Iran

**Keywords:** Melanoma, Cold atmospheric plasma, Doxorubicin, Cell viability, Cytotoxicity, Cancer, Cell biology, Drug discovery, Immunology, Molecular biology, Diseases

## Abstract

Melanoma is responsible for the majority of skin cancer deaths, but there are ways to combat this deadly disease. One method is using anti-neoplastic agents, such as Doxorubicin (DOX). Unfortunately, DOX can be toxic and may lead to drug resistance. However, researchers are excited about the potential of Cold Atmospheric Plasma (CAP) treatment cancer cells and overcome drug resistance selectively. To better understand the effectiveness of the combination of CAP and DOX on melanoma cell viability, cytotoxicity, and cell death, we conducted a comprehensive evaluation and meta-analysis in this study. 41 studies out of 121 met our inclusion criteria. The pooled analysis found that CAP and DOX combination had a significant effect on cell viability (ES = 6.75, 95% CI 1.65 to 11.85, and I2 = 71%) and cytotoxicity (ES = 11.71, 95% CI 3.69 to 19.73, and I2 = 56%). however, no statistically significant association was found between cell death with combination treatment. Our studies have confirmed that the combined treatment of CAP and DOX has a synergistic effect on reducing cell viability and increasing cytotoxicity in melanoma cells. These results can assist researchers in selecting more effective treatment methods to address melanoma.

## Introduction

Cutaneous melanoma remains a significant public health challenge globally. By 2040, there will be approximately 510,000 new cases and 96,000 deaths due to this disease^1^. In the United States alone, around 100,640 individuals are expected to be diagnosed with melanoma in 2024, and approximately 8,290 are projected to die from it^2^. Unfortunately, metastatic melanoma has a survival rate of only 27%^3^, Early diagnosis and treatment are crucial for the prognosis and survival of primary melanoma, which has a 5-year survival rate of 99%. Treatment for melanoma includes chemotherapy^4^, conventional chemotherapy^5^, and immune checkpoint inhibitors^6^. And small molecules against mutant BRAF^7^. Despite advancements in treatment approaches, tumor heterogeneity limits disease-free survival in patients^7^. Tumor heterogeneity confers varying degrees of resistance and survival advantages. As a result, understanding the biology of tumors is always required, as is the development of novel or improved combination therapeutic approaches.

Cancer prevention’s first line of defense is provided by a variety of anti-neoplastic agents^8^. The majority of these agents cause cell cycle arrest and death by targeting or altering Deoxyribonucleic acid (DNA) synthesis and repair mechanisms^9^. One of the most potent chemotherapeutic agents, doxorubicin, also known as adriamycin, has significant therapeutic activity against numerous cancers. DOX is an anthracycline that causes DNA damage by intercalating DNA base pairs and inhibiting topoisomerase II activity^10^. However, its use is limited due to its toxicity, particularly its cardiotoxicity^11^.

In addition, it has been discovered that plasma or plasma-activated solution also has a good killing effect on drug-resistant cancer cells^12^, so CAP is expected to solve drug-resistance issues with clinical cancer chemotherapy. Recent preliminary studies have confirmed that the combination of plasma jet with the anticancer drug tegafur can effectively improve the inactivation of pancreatic cancer cells. In addition, the excellent synergistic effects of CAP and anticancer drugs can effectively reduce the treatment cycle and cumulative dose administered from a clinical perspective. numerous studies regarding the use of CAP for cancer therapy have shown that the appropriate dosages of CAP treatment can selectively kill cancer cells without causing significant damage to normal cells^13–15^. Numerous reactive oxygen and nitrogen species (ROS/RNS)^16,17^are produced by cold physical plasma (partially ionized gas) and Cells also accumulate ROS therefore excess intracellular ROS causes oxidative damage and further induces programmed cell death^18^.

However, the extent to which CAP and DOX combination therapy can influence the cell viability, death, and cytotoxicity of melanoma is yet to be known. Meta-analysis is a method to collect related studies and provide improved statistical power by combining their results. In this study, we aimed to provide a comprehensive systematic review and meta-analysis of the subgroup analysis such as cell line, plasma gases, and treatment time in melanoma cell viability, death, and cytotoxicity by DOX and CAP treatment together or alone.

## Materials and methods

### Search strategy

In this systematic review and meta-analysis, PubMed, Scopus, Web of Science, EMABSE, and Google Scholar electronic databases were searched up to December 2022 using the following search terms: “Melanoma” OR “Malignant Melanoma” OR “Melanoma Malignant” AND “cold atmospheric plasma” OR “Plasma Gases” OR “Gases Plasma” OR “Cold Plasma” OR “Plasma Cold” OR “Non-Thermal Atmospheric Pressure Plasma” OR “Thermal Plasma” OR “plasma jet” AND “Doxorubicin” OR “DOX”. All references cited were manually scanned to find additional studies. We followed the Preferred Reporting Items for Systematic Reviews and Meta-Analyses (PRISMA) framework. This study was registered in PROSPERO (PROSPERO ID: CRD42018117203).

### Inclusion and exclusion criteria

Study selection was performed by two reviewers independently. First, titles and abstracts of all studies were perused to include all the studies on CAP or DOX treatment in melanoma. Then, the full texts of selected papers were retrieved to assess and extract the relevant data thoroughly. Inclusion criteria were as follows: in-vitro experimental studies^2^, case-control studies^3^ original articles;^4^ English language papers;^5^ sufficient data to calculate effect size (ES) and its 95% CI^6^, melanoma cells from different human or murine lines^7^, Eligible studies to investigate severity, death and cell viability and cytotoxicity must include melanoma cells treated with CAP or DOX or both^8^, with diagnostic methods such as MTT, flow cytometry, and information about death or proliferation and Their vitality and cytotoxicity should be reported separately by cell type and type of treatment. Studies were excluded if they were:^1^ Non-original publications, including editorials, commentaries, and review articles;^2^ duplicated studies;^3^ animal subjects;^4^ studies with incomplete information. in-vitro experimental studies^5^, case-control studies^6^ original articles;^7^ English language papers;^8^ sufficient data to calculate effect size (ES) and its 95% CI^9^, melanoma cells from different human or murine lines^10^, Eligible studies to investigate severity, death and cell viability and cytotoxicity must include melanoma cells treated with CAP or DOX or both^11^, with diagnostic methods such as MTT, flow cytometry, and information about death or proliferation and Their vitality and cytotoxicity should be reported separately by cell type and type of treatment^12^, Clinical trial studies^13^, Articles that did not get the minimum score of the checklist. If the two reviewers could not reach an agreement about the selection of papers, the final decision was made by a third reviewer.

### Data extraction and quality assessment

Information was carefully extracted from all eligible publications independently by two reviewers according to inclusion and exclusion criteria. The following information was extracted from each study: article title, author name, country, year of article publication, cell line type, measurement method, gas type, cell life rate, cytotoxicity rate, cell death, treatment time with CAP, dose Drug use. final results and Newcastle-Ottawa Scale (NOS). The quality of eligible studies was evaluated using NOS on a 0–9 scale.0–3 was classified as low-quality, 4–6 as moderate quality, and ≥ 7 as high-quality. The main characteristics of included studies have been summarized in (Tables 1, 2 and 3).

### Statistical analysis

The I2 index was used to assess significant heterogeneity between studies. If the test result was I2 ≥ 50%, indicating the presence of heterogeneity, the random-effects model was used; otherwise, the fixed-effects model was selected. We evaluated publication bias using Egger’s regression intercept test (*P* < 0.05 was considered significant). The meta-analysis was performed with STATA version 11.1 (Stata Corp, College Station, TX, USA).

## Results

**Fig. 1:**
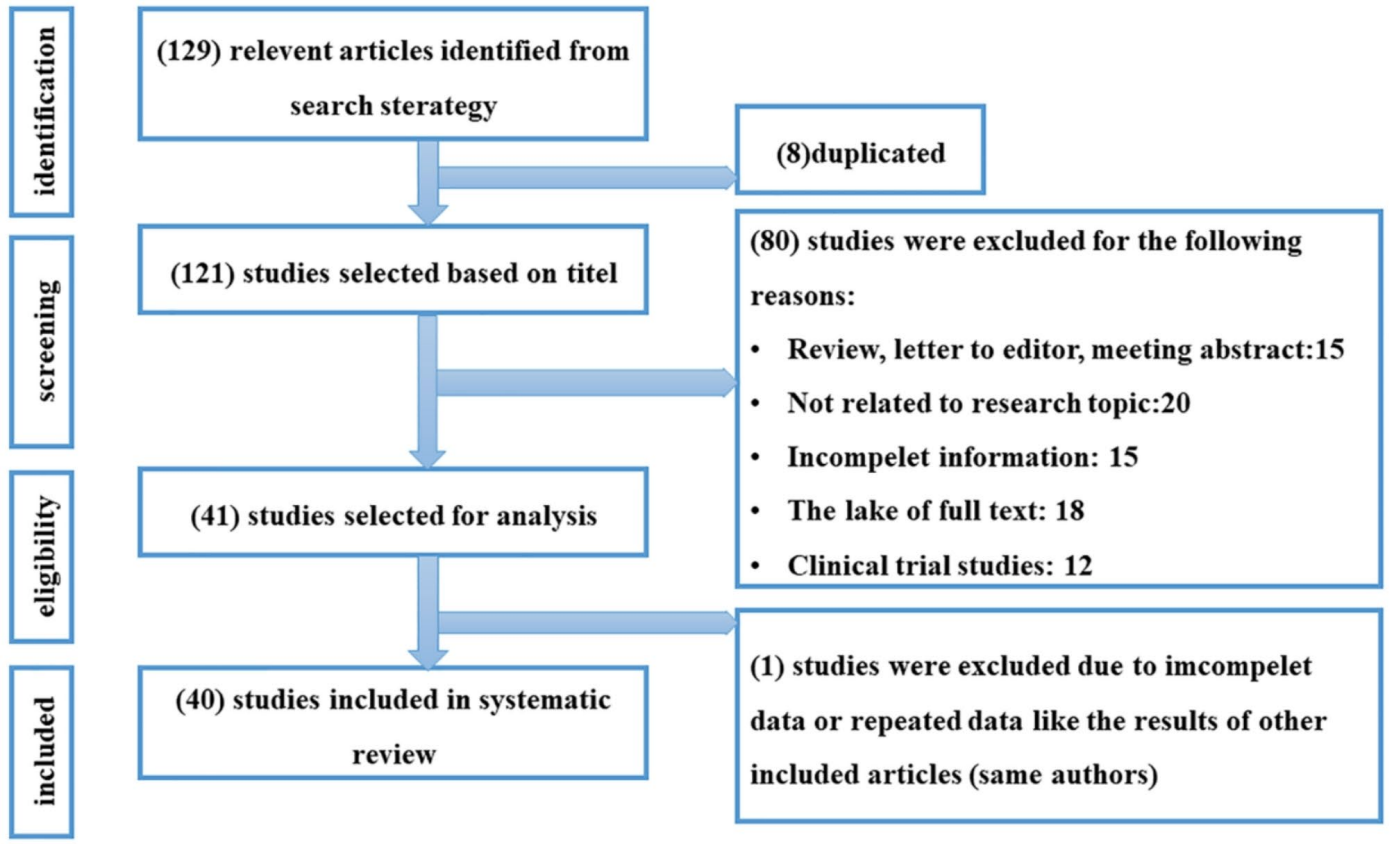
Flow chart for the selection of included studies.

The PRISMA flow diagram of the study is shown in Fig. 1. We found 129 articles related to the title of this study by search strategy, 8 of which were duplicates and were removed. We screened the title and abstract of 121 selected articles for eligibility, and then 80 studies were excluded for non according our inclusion and exclusion criteria. 41 studies have been selected for full-text analysis; after securitization of them, 1 studies were excluded due to the incomplete data or repeated data like the results of other included articles (same authors). Finally^40^, case-control studies were included in the systematic review. Among these selected studies, 20 studies showed the effect of DOX in melanoma, 17 studies investigated the effect of CAP in the treatment of melanoma, and 3 studies investigated the simultaneous relationship between CAP and DOX in melanoma.

The level of cell viability in these studies was mostly measured using (3-(4,5-Dimethylthiazol-2-yl)-2,5-Diphenyltetrazolium Bromide) MTT assay and the cell cytotoxicity was mostly measured by Annexin V flowcytometry, in these studies cell death was mostly measured by assessed (propodium iodide) PI. Egger’s test did non indicate any evidence of publication bias. All included studies were moderate- to high-quality (14 high-quality studies and 26 moderate quality studies). The characteristics of the included studies are described in (Tables 1, 2 and 3).

**Table 1:** Characteristics of the studies included that in the systematic review and meta-analysis.

Author	year	cell line	plasma gas	CNT_viability	CAP_viability	CNT_cell_death	SD-CNT_cell_death	CAP_cell_death	SD_cell_death	CNT_cytotoxicity	SD	CAP_cytotoxicity	SD-cell-death	Test repetition times	NOS
Xu	2017	B16F10	Helium	1	0.4	2	1	78	10	7.3	1.2	5560.00%	230.00%	3	7
Liu	2019	B16F10	Helium	100	57	7	2.1	37.7	2.6	5	0.9	100	12.9	3	8
Saadati	2018	B16F10	Helium	100	47	–	–	–	–	1000	102	13,000	510	3	5
Yan	2018	B16F10	Helium	100	57	–	–	–	–	–		–		3	6
Pefani	2021	B16F10	Helium	100	57.6	1.59	1	2.04	1.5	0.8	0.1	0.7	0.2	3	8
Bekeschus	2017	B16F10	Argon	100	61.5	14.7	3.7	24.7	3.9	–		–		3	7
Lin	2019	B16F10	Argon	100	55	1.5	0.4	4.6	0.7	–		–		2	8
Bekeschus	2020	B16F10	Argon	100	55	3	–	85	–	–		–		3	7
Li	2018	B16F10	Argon	100	78	–		–		–		–		3	6
Gandhirajan	2018	B16F10	Argon	100	90	–		–		0.96	0	2.5	0.3	4	9
Gandhirajan	2018	B16F0	Argon	100	84	–		–		0.94	0	1.4	0.5	4	9
Sagwal	2018	B16F0	Argon	100	82.1	–		–		–		–		3	7
Backer	2022	A375	Argon	100	38	–		–		–		–		3	6
Shaw	2019	A375	Argon	100	40	0	0	39	12.1	2.3	0.6	16	0.5	3	6
Tian	2021	A375	Argon	100	22	–		–		–		–		3	8
Muneekaew	2021	A375	Argon	100	8	–		–		–		–		3	6
Xia	2019	A375	Helium	100	30	–		–		1.6	0.6	54.9	8	3	7
Zhang	2021	A375	Helium	100	93.3	–		–		–		–		3	6
Hasse	2019	SK-Mel-28	Argon	100	40	–		–		–		–		2	8
Hasse	2020	SK-Mel-28	Argon	–	–	–		–		–		–		3	8
Vermeylen	2016	SK-Mel-28	Argon	100	70	–		–		18	–	33	–	3	5
Gandhirajan	2017	SK-Mel-28	Argon	100	70	–		–		0.95	0	7.9	1.2	4	9
Sagwal	2018	SK-Mel-28	Argon	100	89.99	31	10	58.5	6.2	29.5	3.4	35	4	3	7

NOS: Newcastle–Ottawa scale, SD: standard deviation, DOX: doxorubicin, CAP: cold atmospheric plasma, CNT: control.

**Table 2:** Characteristics of the studies included in the systematic review and meta-analysis.

Author	Year	Cell line	Treat time (h)	CNT_viability	DOX_viability	CNT_cell_death	SD-CNT_cell_death	DOX_cell_death	SD-cell-death	CNT_cytotoxicity	SD	DOX_ cytotoxicity	SD	Test repetition times	NOS
Maghsoudinia	2022	B16F10	2.5	100	67	0	0	50	7.2	3	0.5	65	9.8	3	7
Chen	2020	B16F10	6	100	23	–		–		3	1.3	24	4.2	2	5
Jones	2011	B16F10	72	100	58					–		–		3	6
Yang	2022	B16F10	12	–	–	2	0.8	23	5.4	–		–		3	8
Kao	2018	B16F10	24	100	9	–		–		–		–		4	4
Alizadeh	2018	B16F10	48	100	35	–		–		–		–		3	6
An	2021	B16F10	72	100	2.3	–		–		–		–		3	7
Zhu	2010	B16F10	24	100	0	–		–		–		–		2	5
Talelli	2010	B16F10	72	100	9	–		–		–		–		3	4
Banstola	2021	B16F10	24	100	41	0	0.2	48	5.4	–		–		3	8
Mittal	2014	B16F10	48	–	–	11	1.2	54	3.1	–		–		2	6
Park	2008	B16F10	24	–	–	3	1	35	3.4	–		–		3	6
Pefani	2021	B16F10	48	–	–	1.59	1	5.15	1.35	0.8	0.1	4.96	1		8
Patras	2021	B16F10	24	100	15	–		–		–		–		3	5
Grabowska	2021	A375	24	100	61	–		–		–		–		3	6
Lima	2022	A375	48	100	22	9	7.4	27	7.5	–		–		4	7
Salvador	2021	A375	72	100	4.5	7	0.6	45	4.6	–		–		3	6
Pegoraro	2013	A375	24	100	3	–				–		–		3	6
Yu	2019	A375	24	100	28	5.3	0.9	62.1	2.6	–		–		3	9
Lai	2021	A375	24	100	54	0	0	30	0.5	–		–		4	5
Song	2021	A375	24	100	50	–		–		–		–		3	6
Yu	2019	SK-Mel-28	24	100	30	5	0	77	0	–		–		3	9
Song	2021	SK-Mel-28	24	100	30	–		–		–		–		4	6
Sagwal	2018	SK-Mel-28	6	100	40.8	31	10	b	32.3	29.5	3.4	51.7	3.35	3	7

**Table 3:** Characteristics of the studies included that in the systematic review and meta-analysis.

Author	Year	Cell line	Plasma gas	DOX treat time(h)	CNT_viability	CAP_viability	DOX_viability	CAP + DOX_viability	CNT_Cell_death	SD	CAP_Cell_death	SD	DOX_Cell_death	SD
Zhang	2021	A375	Helium	24	100	93.3	63.05	0.012						
Pefani	2021	SKMEL2	Helium	48	100	53.7	57.6	19.3						
Pefani	2021	B16F10	Helium	48	100	57.6	69.3	37.3	1.59	1	2.04	1.5	5.15	1.35
Sagwal (a)	2018	B16F10	Argon	6	100	83.8	11.57	3.5	2.85	0.6	4.74	1.8	1	0.3
Sagwal (b)	2018	B16F0	Argon	6	100	82.1	14.33	7.9						
Sagwal (c)	2018	SK-Mel-28	Argon	6	100	89.99	40.8	13.52	31	10	58.5	6.2	153.3	32.3

Author	Year	CAP + DOX_Cell_death	SD	CNT_cytotoxicity	SD	CAP_cytotoxicity	SD	DOX_cytotoxicity	SD	DOX + CAP_cytotoxicity	SD	Test repetition times	NOS	
Zhang	2021											3	6	
Pefani	2021											3	8	
Pefani	2021	10.22	4.1	0.8	0.1	0.7	0.2	4.96	1	10.4	0.5	3	8	
Sagwal (a)	2018	23.16	6.4	0	0	33.25	9.3	48.27	5.3	86.15	10	3	7	
Sagwal (b)	2018											3	7	
Sagwal (c)	2018	37.51	480.4	29.5	3.4	35	4	51.7	3.35	67.3	6.8	3	7	

### Main results of meta-analysis

#### Meta-analysis of the association between the melanoma cell viability and treatment

**Fig. 2:**
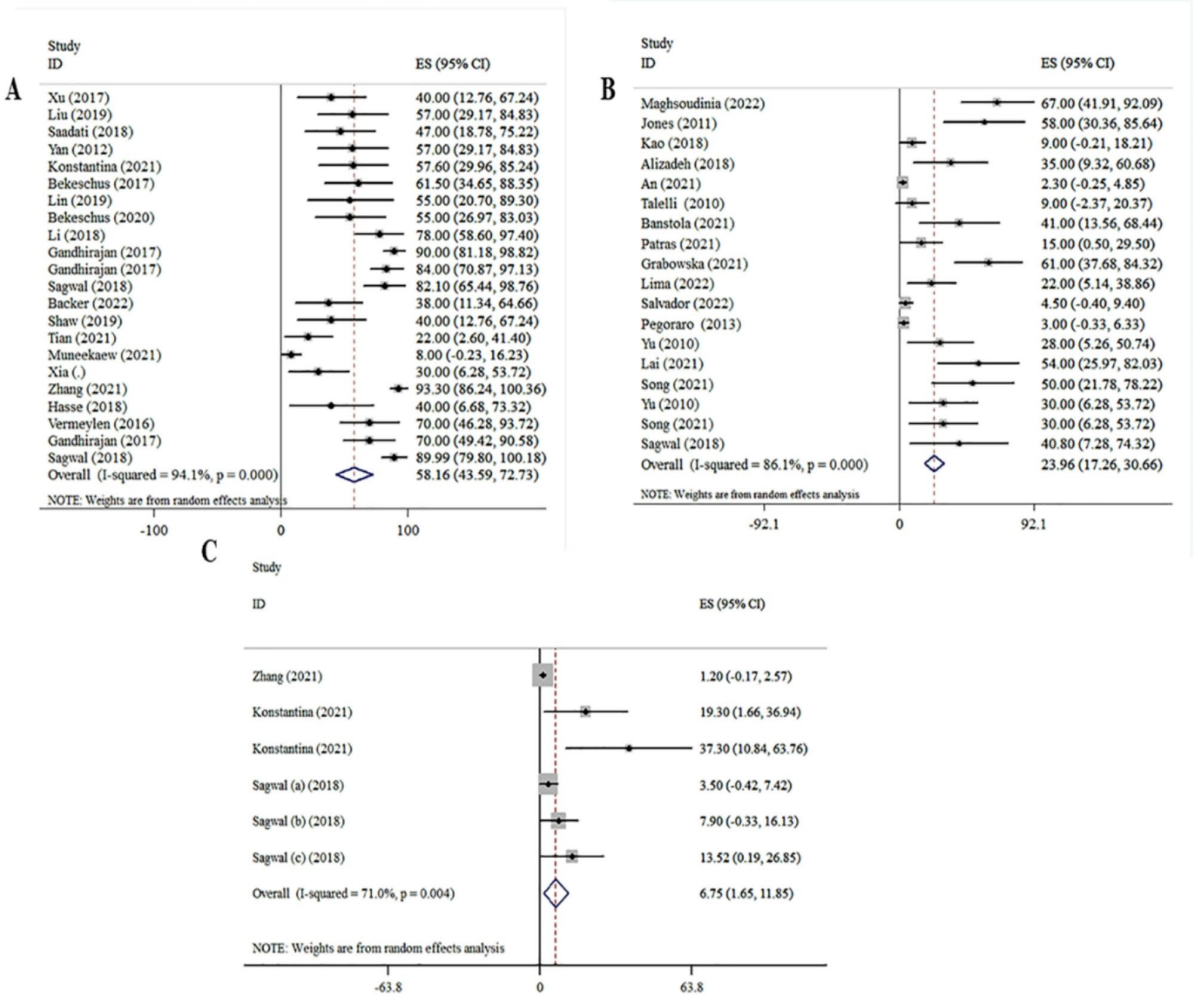
Plot for the association between melanoma treatment and cell viability. (**A**) CAP treatment vs. viability analysis. (**B**) DOX treatment vs. viability analysis. (**C**) DOX-CAP treatment vs. viability.

Pooled analysis of the 25 studies^19–38^, investigated the association between the melanoma cell viability and CAP treatment. The analysis of the dominant model indicated that a significant association existed between melanoma CAP treatment and a decrease the melanoma cell viability ([ES] = 58.16, 95% [CI]: 43.59 to 72.73, I2 = 94.1%). Moreover, the 18 studies^29,39–56^, investigated the association between melanoma cell viability and DOX treatment. The analysis of the dominant model indicated that a significant association existed between melanoma DOX treatment and a decrease the melanoma cell viability ([ES] = 23.96, 95% [CI]: 17.26 to 13.66, I2 = 86.1%). Based on the 6 studies^23,29,35^, investigated the association between melanoma cell viability and CAP and DOX treatment. The analysis of the dominant model indicated that a significant association existed between melanoma CAP and DOX treatment and decreased melanoma cell viability ([ES] = 6.75, 95% [CI]: 1.65 to 11.85, I2 = 71%) (Fig. 2).

#### Meta-analysis of the association between melanoma cell death and treatment

**Fig. 3:**
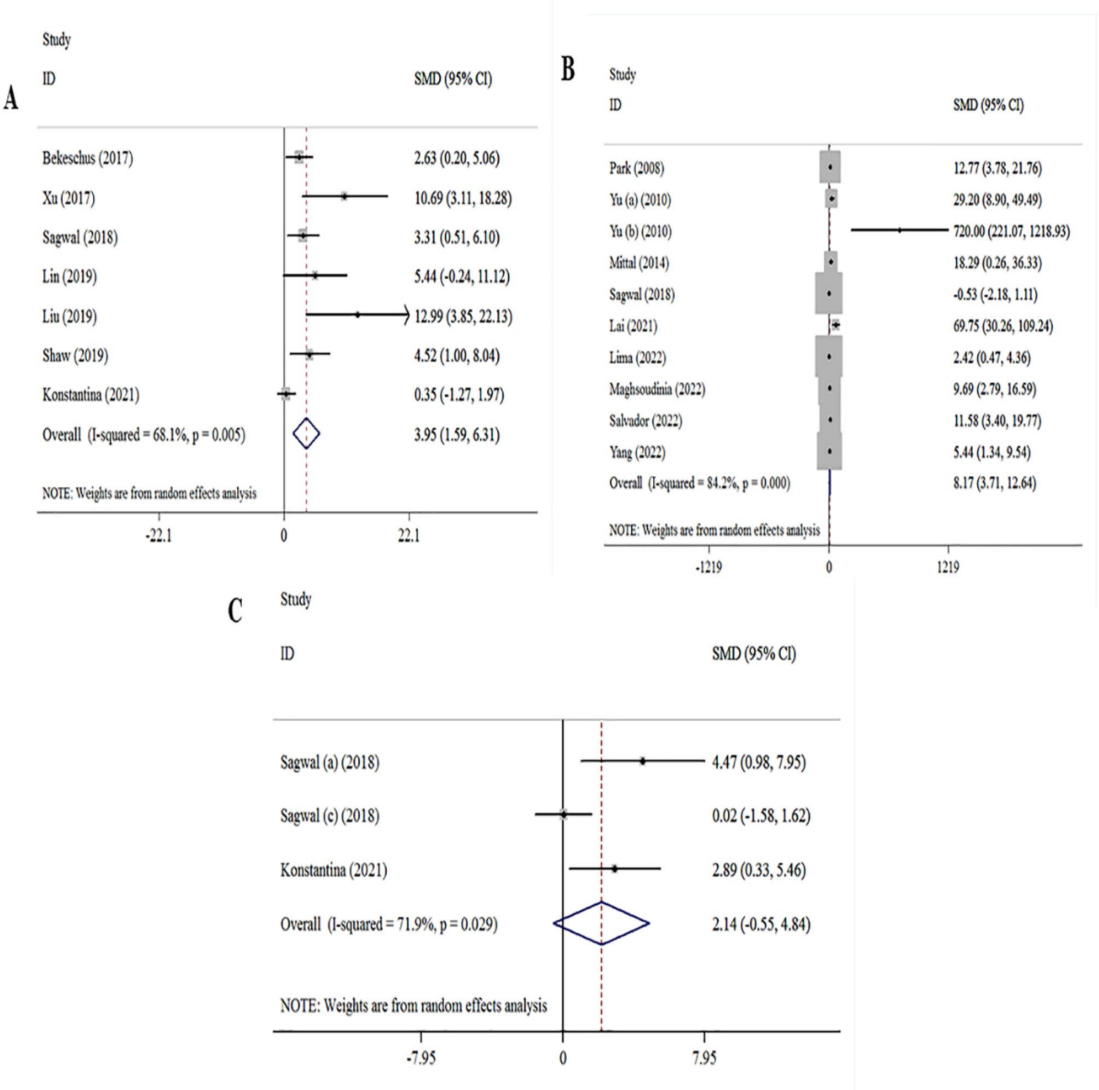
Forest plot of for association between melanoma treatment and cell death. (**A**) CAP treatment vs. cell death analysis. (**B**) DOX treatment vs. cell death analysis. (**C**) DOX-CAP treatment vs. cell death.

7 studies^19,20,23–26,29,31^, evaluated the association between melanoma cell death and CAP treatment. The overall results showed that a significant association existed between melanoma CAP treatment and an increase the melanoma cell death ([ES] = 3.95, 95% [CI]: 1.59 to 6.31, I2 = 68.1%). Moreover, based on 10 studies^23,29,42,48,51,52,54,57,58^ association of melanoma cell death with DOX treatment was evaluated. Significant associations were found for DOX treatment and increased the melanoma cell death ([ES] = 8.17, 95% [CI]: 3.71 to 12.64, I2 = 84.2%). Pooled analysis of the 3 studies^23,29^, investigated the association between melanoma cell death and CAP and DOX treatment. The analysis of the dominant model indicated that an association existed between melanoma CAP and DOX treatment and an increase the melanoma cell death ([ES] = 2.14, 95% [CI]: -0.55 to 4.84, I2 = 71.0%) (Fig. 3).

### Meta-analysis of the association between melanoma cell cytotoxicity and treatment

**Fig. 4:**
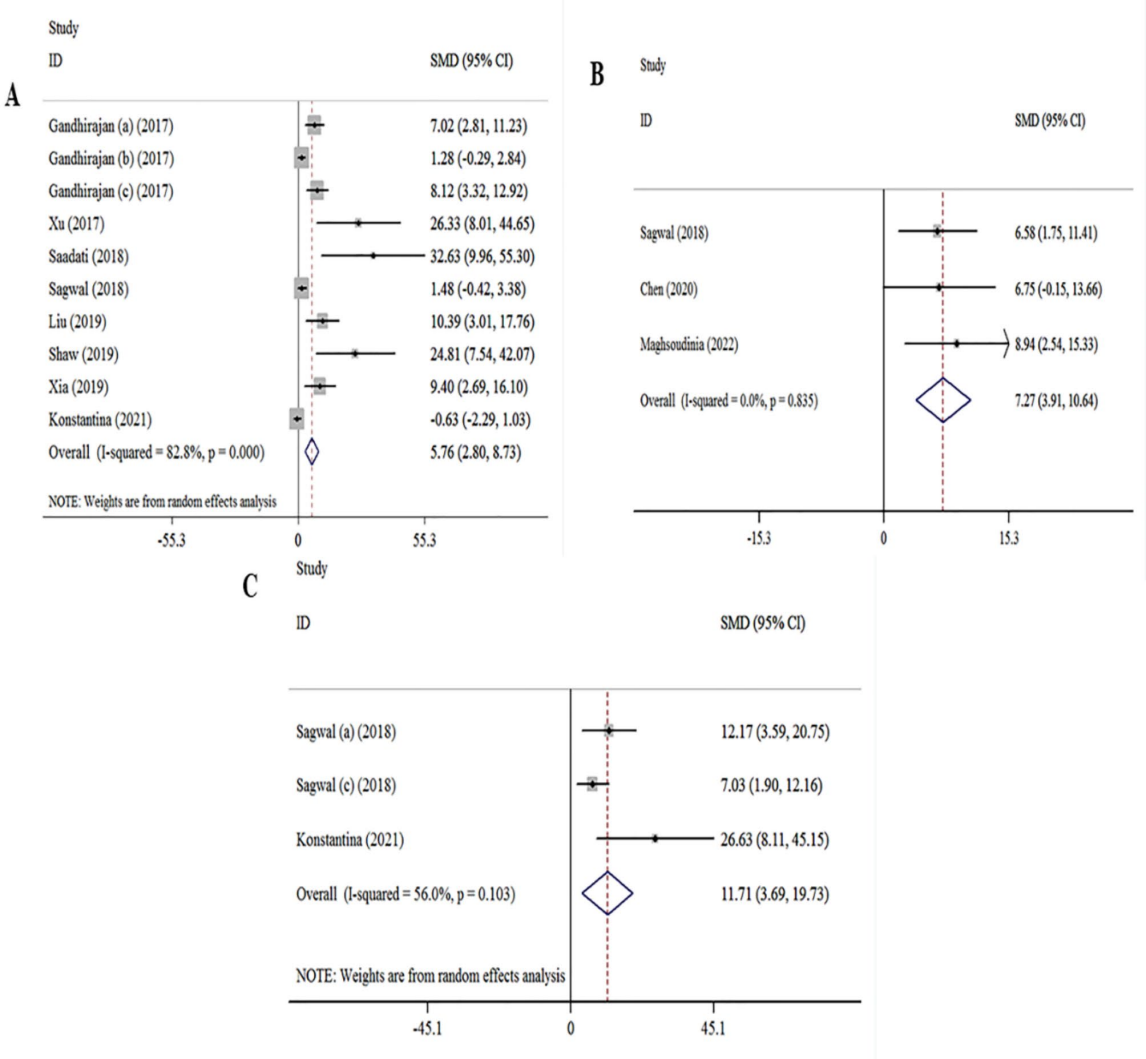
Forest plot of for association between melanoma treatment and cell cytotoxicity. (**A**) CAP treatment vs. cell cytotoxicity analysis. (**B**) DOX treatment vs. cell cytotoxicity analysis. (**C**) DOX-CAP treatment vs. cell cytotoxicity.

Based on 12 studies^19–21,23,28,29,31,34,38^, association of melanoma cell cytotoxicity with CAP treatment was evaluated. Significant associations were found for CAP treatment and increased the melanoma cell cytotoxicity ([ES] = 5.76, 95% [CI]: 2.80 to 8.73, I2 = 82.8%). In addition, no significant association was found between the melanoma cell cytotoxicity and DOX^23,29,39,40^ treatment ([ES] = 7.27, 95% [CI]: 3.91 to 10.64, I2 = 0). 3 studies^23,29^, evaluated the association between the melanoma cell cytotoxicity and CAP and DOX treatment. The overall results showed that a significant association existed between melanoma CAP and DOX treatment and an increase the melanoma cell death ([ES] = 11.71, 95% [CI]: 3.69 to 19.73, I2 = 56%) (Fig. 4).

### Subgroup and intragroup analyses

**Fig. 5:**
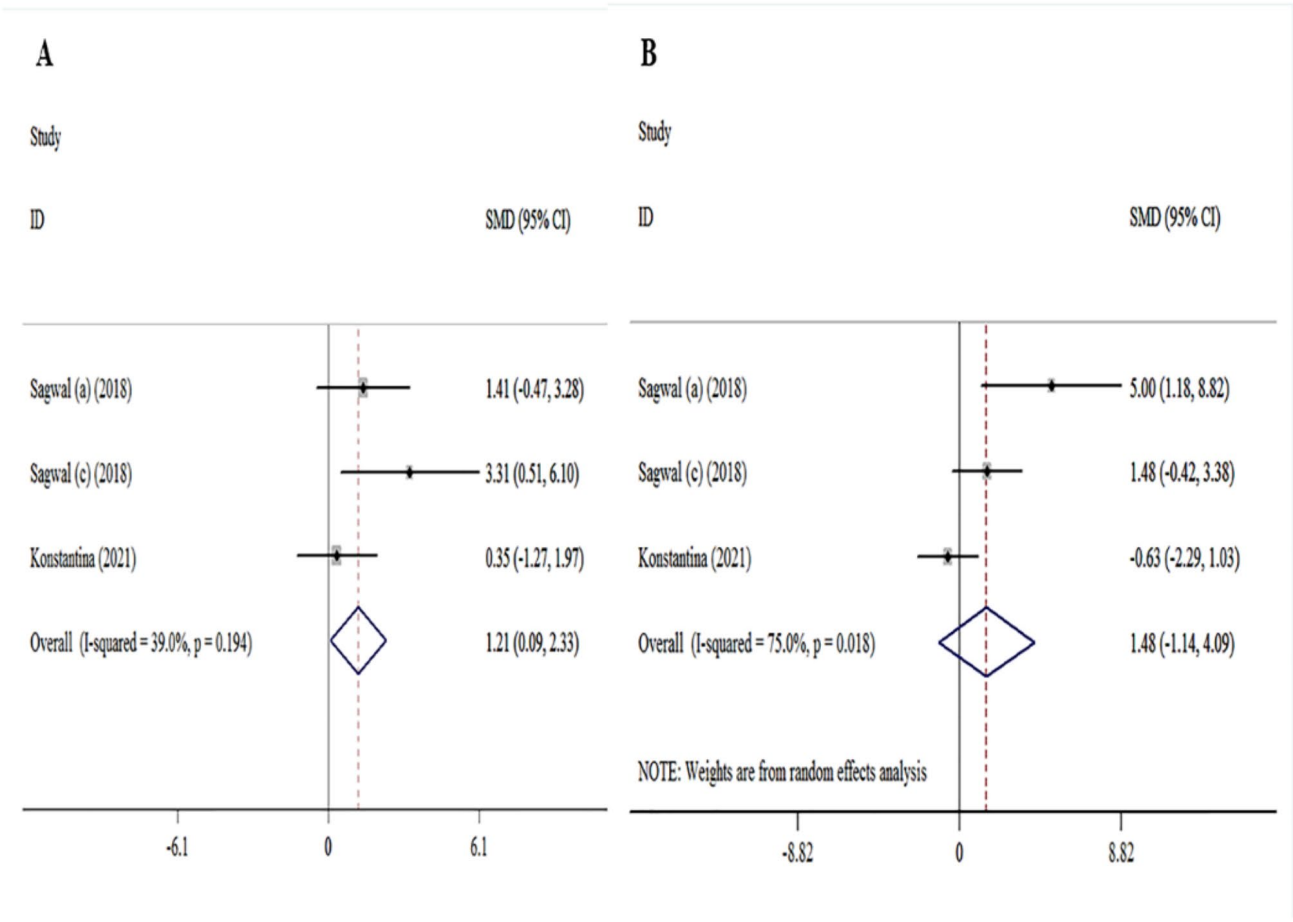
Forest plot of intragroup analysis of the cell death and cytotoxicity in melanoma-based CAP treatment.

**Fig. 6:**
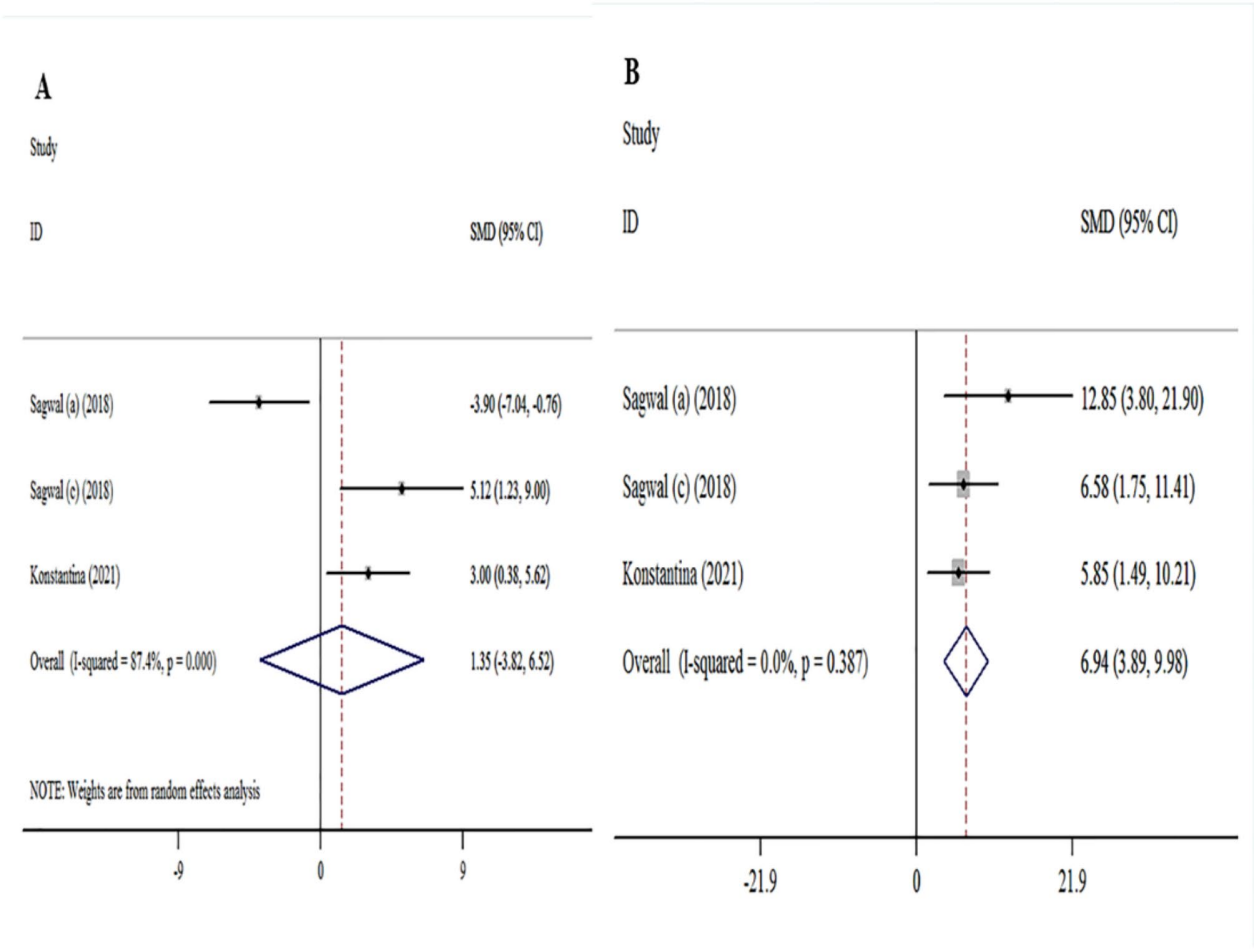
Forest plot of intragroup analysis of the cell death and cytotoxicity in DOX treated melanoma.

Due to significant heterogeneity between studies, subgroup analyses were performed. The data related to the subgroup analyses of the studied studies are shown in (Table 4). In addition, among the 3 studies that investigated the effects of CAP and DOX and used 5 different types of melanoma, intragroup analyses were performed. The analysis of the dominant model indicated that a significant association existed between melanoma cell viability with CAP treatment ([ES] = 82, 95% [CI]: 72.8 to 92.29, I2 = 62.7%) and DOX treatment ([ES] = 40.80% [CI]: 19.63 to 61.97, I2 = 85.6%). intragroup analyses indicated that CAP treatment ([ES] = 1.21% [CI]: 0.09 to 2.33, I2 = 39%) and DOX treatment ([ES] = 1.35% [CI]: -3.82 to 6.52, I2 = 87.4%) have an association with melanoma cell death. Moreover among the 3 studies Significant associations were found for CAP treatment and increased melanoma cell cytotoxicity ([ES] = 1.84% [CI]: -1.14 to 4.09, I2 = 75%) (Figs. 5 and 6).

**Table 4:** Subgroup analysis of the cell viability of CAP and DOX combination in melanoma based on the type of cell line, gas and treatment time.

Treatment	Group	Subgroup	Viability_I2	Viability_ ES
CAP	Cell line	B16F10	70.3	48.43
		B16F0	0	72.96
		A375	98	1.54
		SK-Mel-28	72	52.23
	Gas	Helium	88.5	55.50
		Argon	94.9	59.38
DOX	Cell line	B16F10	87.7	12.18
		A375	86.6	13.63
		SK-Mel-28	0	17.17
	Treat-time	48 h	0	25.92
		24 h	85.8	29.09
		6 h	0	40.80

CAP-DOX treatment	Subgroup	Viability_I2	Viability_ ES	
Cell line	B16F10	83.7	17.76	
	B16F0	0	7.90	
	A375	0	1.20	
	SK-Mel-28	0	13.52	
Gas	Helium	82.0	16.09	
	Argon	23.3	5.71	
Treat-time (h)	48	18.8	25.49	
	24	0	1.20	
	6	23.3	5.71	

## Discussion

Over the past 30 years, melanoma rates have increased worldwide^59^. Tumor heterogeneity, which refers to the genetic and phenotypic differences between tumor cells, can restrict the disease-free survival period for patients^60^, Despite advancements in cancer treatment, novel combination therapies are often required. Recently, there has been significant attention paid to the clinical application of CAP in cancer therapy^61–63^. The ROS generated by CAP plays a significant role in this specific anticancer effect^64–66^. Cancer cells produce a large amount of ROS due to their abnormally active metabolism during rapid proliferation^67^. Injection of only a small dose of CAP generates exogenous reactive species, allowing cancer cells to reach a lethal ROS threshold quickly while normal cells remain unharmed^68^. Additionally, clinical cancer chemotherapy is expected to address drug resistance issues with CAP^12^. As a result, CAP and DOX combination therapy is one of the most effective melanoma chemotherapies that have a positive impact^11,59^. DOX, similar to CAP, is known to generate ROS through the redox cycle, which exacerbates oxidative damage. The synergistic effects of these compounds may; overcome the antioxidant defense mechanisms of cancer cells, induce lipid peroxidation and protein oxidation, and cause mitochondrial dysfunction, ultimately leading to cell death^69,70^. It is non yet clear how effective the combination of CAP and DOX therapy is in influencing the cell viability, death, and cytotoxicity of melanoma. To gain a better understanding of this therapy’s mechanism, we conducted a systematic review and meta-analysis to summarize the role of CAP and DOX therapy in the viability, cytotoxicity, and death of melanoma cells.

The results of previous studies have shown that the treatment of melanoma cells with CAP in the process of dealing with the tumor reduces cell viability^19,20,31^. In addition, past studies have shown that the use of DOX for the treatment of melanoma is effective and reduces cell viability^39,40,45^. The results of our meta-analyses have also confirmed this decrease in cell viability with CAP(ES: 58.23) and DOX(ES: 23) treatment alone compared to the control group. The cell viability test results from the previous study also showed that the combination mode of CAP and DOX was most effective against cancer^23,29^. The results of our analyzes were also in following with previous studies and showed that, the cell viability in the studies that used the combined treatment CAP and DOX was significantly reduced (ES: 6.75) compared to the studies that used the treatment CAP(ES: 58.23) or DOX(ES: 23) alone (Fig. 2). In the subgroup analysis of cell viability in the subgroup of cell line A375, the cell viability was significantly reduced with the combined treatment(ES: 1.20) compared to the CAP(ES: 1.54) or DOX(ES: 13.63) group alone. In addition, in the subgroup of cell line SKMEL28(ES: 13.52) and B16F0(ES: 7.90) the cell viability was significantly reduced with the combined treatment compared to the CAP or DOX group alone. the Treat-time subgroup analysis indicated that the cell viability in the combined treatment of CAP and DOX was significantly reduced(ES: 1.20) compared to group DOX(ES: 29.09) alone. Moreover in the subgroup of the gas used in the CAP device, in the Helium gas group, the cell viability in the combined treatment of CAP and DOX was significantly reduced(ES: 16.09) compared to group CAP(ES: 55.50) alone (Table 4). In the intragroup analyses among the studies that used the combined treatment, following our previous analyses, a significant decrease in cell viability was shown in the combined treatment(ES: 6.75) compared to the CAP(ES: 52.54) and DOX(ES: 40.80) treatment alone (Figs. 5 and 6).

Previous studies have shown that the use of CAP to treat melanoma has increased cytotoxicity^19,20,29^. In addition, previous studies have shown that dox treatment increased cytotoxicity in melanoma cells^23,29,39^. Our analysis in the field of cytotoxicity showed that the combined treatment of CAP and DOX significantly increases (ES: 11.71) cytotoxicity compared to the treatment of CAP(ES: 5.75) or DOX(ES: 7.27) alone (Fig. 4). In addition, in the intragroup analyses among the studies that used the combined treatment, an increase in cytotoxicity was shown in the combined treatment (ES: 11.71) compared to the CAP(ES: 1.48), DOX(ES: 6.94) treatment, but this increase was nonsignificant (Figs. 5 and 6). The results of previous studies, consistent with the results of our analysis, have shown an increase in cytotoxicity due to combined treatment with CAP and DOX^23,29^.

Many studies about the use of CAP for cancer therapy have shown that plasma can initiate cell death^19,20,29^. Similarly, doxorubicin can also cause increased cell death in melanoma cancer cells^23,29,39^. Moreover, a previous study indicated that combined treatment with CAP and DOX had an increased effect on melanoma cell death^23,29^. In contrast, our meta-analyses have shown that cell death is reduced by combined treatment, but this reduction was not significant (Fig. 3). However, in the intra-group analysis of studies that had combined treatment, cell death increased (ES: 2.14) compared to the cap (ES: 1.21) and dox(ES: 1.35) treatment group alone. but, this increase was not statistically significant. As a result, it is not possible to give a firm opinion on this matter (Figs. 5 and 6).

Although the overall results and subgroup analyses were not statistically significant, the inverse association with cell death observed in the overall meta-analysis and subgroup analyses may be linked to variations in cell lines, drug dosages, or experimental conditions in each study.

In the present study, a significant relationship between cell viability and cytotoxicity was observed with the combination of CAP and DOX in melanoma. Furthermore, due to limitations such as high heterogeneity in several included studies, probably the result of variation in study design, and relatively small sample size, further studies are necessary to understand the effect of combined CAP and DOX treatment on cell death.

## Conclusion

Our findings demonstrate that the combination of cold atmospheric plasma (CAP) and doxorubicin (DOX) produces synergistic effects in reducing melanoma cell viability and increasing cytotoxicity compared to either treatment alone. These effects are likely mediated through mechanisms such as enhanced oxidative stress and DNA damage. However, the reliance on in vitro data underscores the need for further in vivo and clinical research to validate these results and to explore the therapeutic potential of this combination in melanoma treatment.

## Limitation

Sources of variation contributing to the high heterogeneity in our study include study design, sample size, and methodology. Suggested future approaches to minimize heterogeneity involve classifying studies based on experimental context. In addition, most studies were in vitro, and validation of in vivo models and clinical trials is needed in future studies.

## Data Availability

Data is provided within the manuscript or supplementary information files.

## Abbreviations

DNA: Deoxyribonucleic acid
DOX: Doxorubicin
CAP: Cold atmospheric plasma
ROS/RNS: Reactive oxygen and nitrogen species
NOS: Newcastle–Ottawa Scale
MTT: 3-(4,5-dimethylthiazol-2-yl)-2,5-diphenyltetrazolium bromide
PI: Propodium iodid
