# Author Correction: Synergistic effects of cold atmospheric plasma and doxorubicin on melanoma: A systematic review and meta-analysis

**DOI:** 10.1038/s41598-025-26577-x

**Published:** 2025-11-04

**Authors:** Zeinab Rostami, Reza Alizadeh-Navaei, Monireh Golpoor, Zahra Yazdani, Alireza Rafiei

**Affiliations:** 1 Department of Immunology, School of Medicine, Mazandaran University of Medical Sciences, KM 18 Khazarabad Road, KhazarSq, Sari, Iran; 2 Student Research Committee, School of Medicine, Mazandaran University of Medical Sciences, Sari, Iran; 3 Gastrointestinal Cancer Research Center, Mazandaran University of Medical Sciences, Sari, Iran

Correction to: *Scientific Reports* 10.1038/s41598-025-90508-z, published online 06 March 2025

This original version of this Article contained errors. At the time of the preparation of this manuscript, the Authors were working on a separate manuscript. As a result of an error during the editing process, text from the Conclusion section of the other paper was used in the Conclusion section of this Article. This has now been updated and the Conclusion section,

“It appears that this is the first meta-analysis to investigate the impact of checkpoint inhibitor treatment (Anti-PD1, Anti-CTLA4) on the level of T-lymphocyte (CD4+, CD8+). Our study found that the response or nonresponse to checkpoint inhibitor treatment in melanoma patients is correlated with the level of T-lymphocyte (CD4+, CD8+). Furthermore, in a melanoma mouse model, tumor volume and survival were found to be linked to T-lymphocyte (CD4+, CD8+) levels following checkpoint inhibitor treatment. These findings may aid researchers in selecting a more appropriate treatment protocol when dealing with melanoma. Our findings demonstrate that the combination of CAP and DOX produces synergistic effects on reducing melanoma cell viability and increasing cytotoxicity. These effects are likely mediated through mechanisms such as enhanced oxidative stress and DNA damage. However, the reliance on in vitro data underscores the need for additional research in vivo and clinical settings to validate these findings.”

now reads:

“Our findings demonstrate that the combination of cold atmospheric plasma (CAP) and doxorubicin (DOX) produces synergistic effects in reducing melanoma cell viability and increasing cytotoxicity compared to either treatment alone. These effects are likely mediated through mechanisms such as enhanced oxidative stress and DNA damage. However, the reliance on in vitro data underscores the need for further in vivo and clinical research to validate these results and to explore the therapeutic potential of this combination in melanoma treatment.”

The original Article has been corrected.

